# Sex-related differences in postoperative complications after pelvic exenteration and their relationship with surgical invasiveness in a multicenter study

**DOI:** 10.1007/s00595-026-03415-9

**Published:** 2026-07-15

**Authors:** Kei Kimura, Masataka Igeta, Yusuke Yatabe, Yuko Fukumoto, Kazuma Ito, Ayako Imada, Jihyung Song, Ryuichi Kuwahara, Yuki Horio, Motoi Uchino, Yusuke Kinugasa, Masataka Ikeda

**Affiliations:** 1 Division of Lower GI, Department of Gastroenterological Surgery, Hyogo Medical University, 1-1, Mukogawa, Nishinomiya, 663-8501 Hyogo Japan; 2 Department of Biostatistics, Hyogo Medical University, Nishinomiya, Hyogo Japan; 3 Department of Gastrointestinal Surgery, Institute of Science Tokyo, Tokyo, Japan; 4 Division of Inflammatory Bowel Disease Surgery, Department of Gastroenterological Surgery, Hyogo Medical University, Nishinomiya, Japan

**Keywords:** Pelvic exenteration, Rectal cancer, TME, Sex difference

## Abstract

**Purpose:**

To investigate the sex-related differences in postoperative complications following pelvic exenteration.

**Methods:**

This retrospective subanalysis used a multicenter registry of patients who underwent pelvic exenteration for locally advanced or recurrent rectal cancer. We included 218 patients (177 men and 41 women) in this study. The sex-related differences in postoperative complications were evaluated, and sex-by-factor interactions were assessed using logistic regression models, including sex, each perioperative factor, and the corresponding interaction term.

**Results:**

A significant sex-by-surgical approach interaction was observed for Clavien–Dindo Grade II or higher complications (*p*_interaction_ < 0.01). In open surgery, Grade II or higher complications occurred in 82.7% (43/52) of men compared with 38.5% (5/13) of women, whereas no clear sex difference was observed in minimally invasive surgery (69.6% [87/125] vs. 75.0% [21/28]). No significant sex-by-surgical approach interaction was detected for grade III or higher complications. Conversely, a significant sex-by-operative time interaction was observed, which was not apparent for Grade II complications.

**Conclusion:**

Sex-related differences in postoperative complications after pelvic exenteration were not uniform but emerged with increased surgical invasiveness. Therefore, sex may affect the postoperative risk under conditions of increased surgical stress.

**Supplementary Information:**

The online version contains supplementary material available at https://doi.org/10.1007/s00595-026-03415-9.

## Introduction

Pelvic exenteration (PE) is a highly invasive but potentially curative surgical option for patients with locally advanced or recurrent rectal cancer (LARC or LRRC). However, the pursuit of oncological clearance through PE is accompanied by substantial morbidity, with overall complications occurring in up to 50% of patients and major complications in 15%–35% of patients [1, 2].

The UK Pelvic Exenteration Network lexicon classifies PE into conventional and high-complexity PE, with the latter encompassing pelvic sidewall excisions or bony pelvic resections, such as sacrectomy or pubic bone resection [3]. Although high-complexity PE is associated with longer operative times and greater blood loss, accumulating evidence suggests that this technically demanding procedure is not consistently associated with higher morbidity than conventional PE [4, 5]. This finding likely reflects advances in multidisciplinary team management, centralization of PE in high-volume centers, and progress in reconstructive techniques and preoperative therapies [6–9]. Consequently, recent research has predominantly focused on procedure-related complexities, such as the effects of lateral compartment involvement and bony pelvic resection on surgical invasiveness and outcomes [10].

In contrast, patient-related factors, particularly sex-related differences, have received much less attention in studies on PE. Male and female pelvic anatomy markedly differ in pelvic width, depth, and visceral fat distribution, which can directly influence technical difficulty, bleeding risk, and postoperative complications. However, the translation of these sex-related differences into postoperative outcomes after PE has not been well defined [11].

We previously reported the results of a large Japanese multicenter study demonstrating the safety and feasibility of robot-assisted PE for advanced and recurrent pelvic malignancies [12]. Using this multicenter dataset, this study aimed to investigate sex-related differences in postoperative complications following PE, with particular attention to the interactions between sex and perioperative factors.

## Materials and methods

### Study design

This study was a retrospective subanalysis of a multicenter registry from Yatabe et al. titled “*Robotic versus open and laparoscopic pelvic exenterations for pelvic cancer: a multicenter propensity-matched analysis in Japan*” [12]. The original registry included patients who underwent PE between January 2012 and October 2022 at 26 institutions. We extracted LARC and LRRC cases from this dataset to assess sex-related differences in postoperative complications. Ethical approval was obtained from the Institutional Review Board of the Hyogo Medical University (IRB code: 5095). All data were fully anonymized and retrospectively obtained. Therefore, individual informed consent was waived, and opt-out consent was obtained in accordance with institutional guidelines. The investigators of the present analysis were not involved in the original data collection process.

### Study population

The inclusion and exclusion criteria were identical to those of the original registries. Patients who underwent PE for LARC or LRRC were eligible for inclusion, while those with non-colorectal malignancies were excluded from the study. Of the 261 patients in the registry, 218 met the eligibility criteria for the present analysis, comprising 177 men and 41 women.

### Definitions

PE was categorized as follows: (1) total PE, which involved en bloc removal of the rectum and genitourinary organs, including the lower ureters, bladder, and internal reproductive organs; (2) anterior PE, which was performed as a supralevator procedure　involving the en bloc resection of the bladder and reproductive organs while sparing the lower rectum, regardless of whether a colorectal anastomosis or a permanent stoma was performed; and (3) posterior PE, which comprised removal of the rectum and reproductive organs while preserving the bladder. R0 resection was defined as the absence of microscopic tumors at the resection margin.

### Data collection

All data used in this study were extracted from the original multicenter study. We collected information on the patients’ background, such as age, sex, body mass index, and American Society of Anesthesiologists physical status. The clinical factors included cancer type (primary vs. recurrent) and the presence or absence of distant metastasis. Preoperative information included the presence or absence of preoperative therapy. The intraoperative data included the surgical approach (open vs. minimally invasive surgery [MIS], such as laparoscopic or robotic), type of PE (total PE vs. anterior PE/ posterior PE), presence or absence of anal preservation, presence or absence of combined bony pelvic excision, presence or absence of perineal reconstruction, operative time, blood loss, and presence or absence of transfusion. The postoperative outcomes were resection margin status and 30-day postoperative complications.

Postoperative complications within 30 days were graded using the Clavien–Dindo classification based on a retrospective chart review at each participating institution.

### Outcome measures

Clavien–Dindo Grade II or higher and Grade III or higher complications were assessed as study outcomes. The patients were stratified by sex, and perioperative variables and postoperative outcomes were compared between male and female patients.

### Statistical analysis

Continuous variables are presented as median with interquartile range [IQR] and categorical variables as number (percentage). Baseline characteristics and perioperative variables were compared between male and female patients using the Mann–Whitney U test for continuous variables and the chi-square test or Fisher’s exact test for categorical variables, as appropriate, using JMP Student edition 18.2.1 software program (SAS Institute, Cary, NC, USA).

To examine whether the associations between perioperative factors and postoperative outcomes differed by sex, we assessed sex-by-factor interactions using logistic regression models for binary outcomes (Grade II or higher complications, Grade III or higher complications), including sex, the factor of interest, and the corresponding sex-by-factor interaction term. These models were used to evaluate the potential effect modification by sex and were not intended to identify independent risk factors for postoperative complications. Continuous variables were categorized using median cutoffs (age: 63 years, body mass index: 21.5 kg/m², operative time: 678 min, and blood loss: 650 mL) for descriptive presentation in the interaction tables. The statistical significance of the interaction effects was evaluated using the *p* value for the interaction term (*p*_interaction_). Interaction analyses were exploratory, and no multiplicity adjustments were applied.

To address potential confounding due to baseline imbalances between male and female patients, additional adjusted interaction analyses were performed for the main interaction results. These models included sex, the factor of interest, the corresponding sex-by-factor interaction term, BMI, primary/recurrent disease, and combined bony pelvis excision. Given the limited number of female patients and events, these adjusted analyses were restricted to the main interaction findings rather than being applied to all exploratory interaction analyses.

To account for potential inter-institutional clustering, sensitivity analyses were performed using generalized estimating equations with institutions as the clustering unit, an independent working correlation structure, and robust sandwich variance estimates. These models were fitted for the main interaction findings and included sex, the factor of interest, the corresponding sex-by-factor interaction term, BMI, primary/recurrent disease, and combined bony pelvic resection.

All tests were two-sided, and a *p* value < 0.05 was considered statistically significant. Analyses were performed using the SAS software program (SAS Institute Inc., Cary, NC, USA).

## Results

### Patients’ characteristics

The patients’ characteristics are presented in Table 1. Male patients had a significantly higher body mass index (*p <* 0.01*)* and more frequently underwent total PE than female patients (*p* < 0.01). Intraoperative blood loss tended to be higher in male than in female patients; however, this difference was not statistically significant (*p* = 0.07).

**Table 1:** Patients’ characteristics

	Male	Female	*p*-value
Age, median (IQR)	63 (54, 70)	62 (51, 69)	0.46
BMI (kg/m^2^), median (IQR)	21.8 (19.5, 23.9)	19.4 (17.5-22.2)	<0.01
ASA-PS, n (%)			
≤2	155 (87.6)	38 (92.7)	0.43
≥3	22 (12.4)	3 (7.3)	
Cancer type, n (%)			
Primary	146 (82.5)	24 (58.5)	<0.01
Recurrent	31 (17.5)	17 (41.5)	
Distant metastasis, n (%)			
Yes	27 (15.3)	7 (17.1)	0.77
No	150 (84.7)	34 (82.9)	
Preoperative therapy, n (%)			
Yes	110 (62.2)	24 (58.5)	0.67
No	67 (37.8)	17 (41.5)	
Surgery type, n (%)			
TPE	137 (77.4)	10 (24.4)	<0.01
APE, PPE	40 (22.6)	31 (75.6)	
Surgical approach, n (%)			
Open	52 (29.4)	13 (31.7)	0.77
Laparoscopic, Robotic	125 (70.6)	28 (68.3)	
Anal preservation, n (%)			
Yes	58 (32.8)	19 (46.3)	0.11
No	119 (67.2)	22 (53.7)	
Combined bony pelvic excision, n (%)			
Yes	15 (8.5)	7 (17.1 )	0.10
No	162 (91.5)	34 (82.9)	
Perineal reconstruction, n (%)			
Yes	115 (65.0)	23 (56.1)	0.29
No	62 (35.0)	18 (43.9)	
Operative time (min), median (IQR)	683 (563-815)	660 (524-806)	0.76
Blood loss (mL), median (IQR)	725 (281-1777)	540 (38-1218)	0.07
Transfusion, n (%)			
Yes	81 (45.8)	15 (36.6)	0.29
No	96 (54.2)	26 (63.4)	
≥ Grade II complication, n (%)			
Yes	130 (73.4)	26 (63.4)	0.20
No	47 (26.6)	15 (36.6)	
≥ Grade III complication, n (%)			
Yes/No	56 (31.6)	12 (29.3)	0.77
No	121 (68.4)	29 (70.7)	
Resection margin, n (%)			
Positive	15 (8.5)	2 (4.9)	0.58
Negative	160 (90.4)	39 (95.1)	
Unknown	2 (1.1)	0	
Postoperative hospital stay, median (IQR)	30 (23-46)	30 (23-40)	0.42
Readmission within 30 days, n			
Yes	10 (5.7)	2 (4.9)	0.85
No	167 (94.4)	39 (95.1)	

BMI, body mass index; IQR, interquartile range; ASA, American Society of Anesthesiologists; TPE, total pelvic exenteration; APE, anterior pelvic exenteration; PPE, posterior pelvic exenteration

### Grade II or higher complications

Sex-by-factor interaction analyses for Grade II or higher complications are shown in Table 2, and effect estimates are provided in Supplementary Table S1. A significant interaction between sex and surgical approach was observed (*p*_interaction_ < 0.01). In open surgery, Grade II or higher complications occurred in 82.7% (43/52) of male patients compared with 38.5% (5/13) of female patients. In contrast, in the MIS group, the grade II or higher complication rate was similar between men and women (69.6% [87/125] vs. 75.0% [21/28]).

**Table 2:** Sex-by-factor interactions analyses of Grade II or higher complications

Variable	Sex	CD grade ≥ II				*p*_interaction_^#^
		No		Yes		
		n	%	n	%	
Age						0.01
Age < 63	Male	22	26.5	61	73.5	
	Female	10	47.6	11	52.4	
Age ≥ 63	Male	25	26.6	69	73.4	
	Female	5	25.0	15	75.0	
BMI						0.22
BMI < 21.5	Male	24	29.6	57	70.4	
	Female	7	26.9	19	73.1	
BMI > 21.5	Male	22	23.2	73	76.8	
	Female	8	53.3	7	46.7	
ASA						0.33
ASA ≤ 2	Male	41	26.5	114	73.5	
	Female	13	34.2	25	65.8	
ASA ≥ 3	Male	6	27.3	16	72.7	
	Female	2	66.7	1	33.3	
Primary or recurrence						0.19
Primary	Male	42	28.8	104	71.2	
	Female	8	33.3	16	66.7	
Recurrence	Male	5	16.1	26	83.9	
	Female	7	41.2	10	58.8	
Distant metastasis						0.09
No	Male	41	27.3	109	72.7	
	Female	13	38.2	21	61.8	
Yes	Male	6	22.2	21	77.8	
	Female	2	28.6	5	71.4	
Preoperative therapy						0.85
No	Male	23	34.3	44	65.7	
	Female	8	47.1	9	52.9	
Yes	Male	24	21.8	86	78.2	
	Female	7	29.2	17	70.8	
Surgery type						0.63
TPE	Male	35	25.6	102	74.5	
	Female	4	40.0	6	60.0	
APE/PPE	Male	12	30.0	28	70.0	
	Female	11	35.5	20	64.5	
Surgical approach						<0.01
Open	Male	9	17.3	43	82.7	
	Female	8	61.5	5	38.5	
Laparoscopic/Robotic	Male	38	30.4	87	69.6	
	Female	7	25.0	21	75.0	
Anal preservation						0.88
No	Male	29	24.4	90	75.6	
	Female	7	31.8	15	68.2	
Yes	Male	18	31.0	40	69.0	
	Female	8	42.1	11	57.9	
Combined bony pelvic excision						0.34
No	Male	46	28.4	116	71.6	
	Female	13	38.2	21	61.8	
Yes	Male	1	6.7	14	93.3	
	Female	2	28.6	5	71.4	
Perineal reconstruction						0.47
No	Male	17	27.4	45	72.6	
	Female	8	44.4	10	55.6	
Yes	Male	30	26.1	85	73.9	
	Female	7	30.4	16	69.6	
Operative time (min)						0.76
< 678	Male	28	32.2	59	67.8	
	Female	8	36.4	14	63.6	
≥ 678	Male	19	21.1	71	78.9	
	Female	7	36.8	12	63.2	
Blood loss (mL)						0.07
< 650	Male	30	35.3	55	64.7	
	Female	6	25.0	18	75.0	
≥ 650	Male	17	18.7	74	81.3	
	Female	9	52.9	8	47.1	
Transfusion						0.39
No	Male	29	30.2	67	69.8	
	Female	9	34.6	17	65.4	
Yes	Male	17	21.0	64	79.0	
	Female	6	40.0	9	60.0	

^#^*p*_interaction_ denotes the *p* value for the interaction term obtained from logistic regression models including sex, the factor of interest, and their interaction. Regarding continuous variables, *p*_interaction_ was calculated using sex and the continuous form of the factor. Categorized results of continuous variables are presented for descriptive and interpretative purposes only. Effect estimates with 95% confidence intervals are provided in Supplementary Table S1

BMI, body mass index; ASA, American Society of Anesthesiologists; TPE, total pelvic exenteration; APE, anterior pelvic exenteration; PPE, posterior pelvic exenteration

### Grade III or higher complications

Sex-by-factor interaction analyses for Grade III or higher complications are shown in Table 3, and effect estimates are provided in Supplementary Table S2. No significant interaction between sex and surgical approach was observed (*p*_interaction_ = 0.53). In open surgery, Grade III or higher complications occurred in 32.7% (17/52) of male patients and 23.1% (3/13) of female patients, whereas in MIS, the corresponding rates were 31.2% (39/125) and 32.1% (9/28), respectively. A significant interaction between sex and operative duration was identified (*p*_interaction_ < 0.01). Among patients with prolonged operative times, Grade III or higher complications occurred more frequently in male than in female patients.

**Table 3:** Sex-by-factor interactions analyses of Grade III or higher complications

Variable	Sex	CD grade ≥ III				*p*_interaction_^#^
		No		Yes		
		n	%	n	%	
Age						<0.01
Age < 63	Male	50	60.2	33	39.8	
	Female	16	76.2	5	23.8	
Age ≥ 63	Male	71	75.5	23	24.5	
	Female	13	65.0	7	35.0	
BMI						0.26
BMI < 21.5	Male	59	72.8	22	27.2	
	Female	17	65.4	9	34.6	
BMI > 21.5	Male	62	65.3	33	34.7	
	Female	12	80.0	3	20.0	
ASA						0.98
ASA ≤ 2	Male	107	69.0	48	31.0	
	Female	27	71.0	11	29.0	
ASA ≥ 3	Male	14	63.6	8	36.4	
	Female	2	66.7	1	33.3	
Primary or recurrence						0.51
Primary	Male	103	70.6	43	29.4	
	Female	17	70.8	7	29.2	
Recurrence	Male	18	58.1	13	41.9	
	Female	12	70.6	5	29.4	
Distant metastasis						0.28
No	Male	101	67.3	49	32.7	
	Female	25	73.5	9	26.5	
Yes	Male	20	74.1	7	25.9	
	Female	4	57.1	3	42.9	
Preoperative therapy						0.55
No	Male	46	68.7	21	31.1	
	Female	13	76.5	4	23.5	
Yes	Male	75	68.2	35	31.8	
	Female	16	66.7	8	33.3	
Surgery type						0.43
TPE	Male	93	67.9	44	32.1	
	Female	8	80.0	2	20.0	
APE/PPE	Male	28	70.0	12	30.0	
	Female	21	67.7	10	32.3	
Surgical approach						0.53
Open	Male	35	67.3	17	32.7	
	Female	10	76.9	3	23.1	
Laparoscopic/Robotic	Male	86	68.8	39	31.2	
	Female	19	67.9	9	32.1	
Anal preservation						0.91
No	Male	76	63.9	43	36.1	
	Female	14	63.6	8	36.4	
Yes	Male	45	77.6	13	22.4	
	Female	15	79.0	4	21.0	
Combined bony pelvic excision						0.39
No	Male	116	71.6	46	28.4	
	Female	25	75.8	9	26.5	
Yes	Male	5	33.3	10	66.7	
	Female	4	50.0	3	42.9	
Perineal reconstruction						0.80
No	Male	46	74.2	16	25.8	
	Female	14	77.8	4	22.2	
Yes	Male	75	65.2	40	34.8	
	Female	15	65.2	8	34.8	
Operative time (min)						<0.01
< 678	Male	68	78.2	19	21.8	
	Female	14	63.6	8	36.4	
≥ 678	Male	53	58.9	37	41.1	
	Female	15	78.9	4	21.1	
Blood loss (mL)						0.09
< 650	Male	63	74.1	22	25.9	
	Female	15	62.5	9	37.5	
≥ 650	Male	57	62.6	34	37.4	
	Female	14	82.4	3	17.6	
Transfusion						0.33
No	Male	66	68.8	30	31.2	
	Female	17	65.4	9	34.6	
Yes	Male	55	67.9	26	32.1	
	Female	12	80.0	3	20.0	

^#^*p*_interaction_ denotes the *p* value for the interaction term obtained from logistic regression models including sex, the factor of interest, and their interaction. Regarding continuous variables, *p*_interaction_ was calculated using sex and the continuous form of the factor. Categorized results of continuous variables are presented for descriptive and interpretative purposes only. Effect estimates with 95% confidence intervals are provided in Supplementary Table S2

BMI, body mass index; ASA, American Society of Anesthesiologists; TPE, total pelvic exenteration; APE, anterior pelvic exenteration; PPE, posterior pelvic exenteration

### Sensitivity analysis

In the adjusted interaction analyses accounting for BMI, primary/recurrent disease, and combined bony pelvic excision, the main interaction findings were consistent. For Grade II or higher complications, the sex-by-surgical approach interaction remained evident after adjustment (*p*_interaction_ = 0.0087). The adjusted OR for open versus minimally invasive surgery was 0.227 (95% CI, 0.051–0.911) in female patients and 2.046 (95% CI, 0.930–4.896) in male patients. For Grade III or higher complications, the sex-by-operative time interaction also remained evident after adjustment (*p*_interaction_ = 0.0078). The adjusted OR per 60-minute increase in operative time was 0.798 (95% CI, 0.614–1.000) in female patients and 1.131 (95% CI, 1.009–1.273) in male patients.

In sensitivity analyses using generalized estimating equations to account for clustering by institution, the sex-by-surgical approach interaction for Grade II or higher complications remained evident (*p*_interaction_ = 0.018). The direction of the sex-by-operative time interaction for Grade III or higher complications was generally consistent with the primary analysis, although the score test was attenuated (*p*_interaction_ = 0.089).

## Discussion

In this study, we examined sex-related interactions in the postoperative complications following PE. The principal finding was that differences in postoperative complications between men and women were not uniform but became apparent under conditions of increased surgical invasiveness. Specifically, regarding Clavien–Dindo Grade II or higher complications, a significant sex-by-surgical approach interaction was observed, with such complications occurring more frequently in men than in women in open surgery, whereas no clear sex difference was detected in MIS. In contrast, no significant difference was observed in Grade III or higher complications according to the surgical approach. This finding should be interpreted cautiously because the number of female patients and Grade III or higher events was limited, resulting in limited statistical power and precision for detecting sex-by-factor interactions. In addition, a significant sex-by-operative time interaction was identified, and consistent directional trends with increasing blood loss were observed for Clavien–Dindo Grade II or higher and Grade III or higher complications.

Taken together, these findings suggest that sex-related differences in postoperative complications may emerge through interactions with increasing surgical stress rather than representing a constant baseline difference between men and women. This interpretation aligns with the objective of the present study, which focused on whether the associations between perioperative factors and postoperative complications differed between men and women. Standard models, including only the main effects, are not designed to directly evaluate effect modification by sex.

Previous studies have identified several factors associated with increased postoperative complications after PE. Among patient-related factors, previous radiotherapy has been reported as a major risk factor, with a complication rate as high as 47% in irradiated patients compared with 23% in non-irradiated patients [13, 14]. Additionally, several procedure-related factors reflecting surgical invasiveness, such as surgery for LRRC, combined pelvic sidewall or bony pelvic resection, prolonged operative time, and extensive blood loss, are associated with a higher risk of postoperative complications [2, 15, 16]. Collectively, greater surgical invasiveness is associated with increased postoperative complications after PE; however, whether these associations differ between men and women is unknown.

The sex-by-surgical invasiveness interaction observed in this study may be partly explained by sex-specific anatomical variations. The male pelvis is generally narrower and deeper than the female pelvis, which restricts the working space for deep pelvic dissection and increases technical difficulty [17]. In rectal cancer surgery, such anatomical constraints are associated with greater procedural complexity and sex-related differences in postoperative complications [18]. Miyake et al. reported that male patients undergoing transanal total mesorectal excision had a longer operative time, greater blood loss, and higher complication rates than female patients, which were attributed to restricted pelvic anatomy [11]. Furthermore, male pelvic anatomy carries a higher risk of conversion from MIS to open surgery, which serves as a surrogate marker for heightened surgical stress and adverse postoperative outcomes [19, 20]. In the setting of PE, where baseline surgical invasiveness is already high, these anatomical constraints may not act as independent risk factors but as amplifiers of surgical stress. Such amplification could contribute to the emergence of sex-related differences in postoperative complications under highly invasive conditions.

Clinically, the main finding of this study was a marked sex-by-surgical approach interaction for Grade II or higher complications, which was observed in open PE but not in MIS. Although analyses of Grade III or higher complications were limited by the small number of events, consistent trends were observed with increasing operative time and blood loss. These findings suggest that sex-related differences in postoperative complications may become more evident under conditions of increased stress. These findings warrant validation in larger and more standardized cohorts.

This study has several limitations. First, this study was an exploratory subgroup analysis based on a retrospective multicenter registry, and the number of female patients and events was limited. Because interaction effects generally require larger sample sizes than main effects, the present study had limited statistical power and precision in detecting sex-by-factor interactions, particularly for Grade III or higher complications with fewer events.

Second, the baseline characteristics were not fully balanced between male and female patients, which may have affected the evaluation of sex-by-factor associations. Although the main interaction findings were generally consistent after adjustment for BMI, primary/recurrent disease, and combined bony pelvic excision, residual confounding due to other measured, unmeasured, or insufficiently measured factors cannot be excluded from this study.

Third, although sensitivity analyses accounting for clustering by institution were performed, inter-institutional variability may not have been fully captured in this study. Therefore, residual center-related effects cannot be ruled out.

Finally, because of the retrospective design and limited event numbers, the identified sex-related interactions should be interpreted with caution and viewed as hypothesis generating rather than conclusive. However, the observed interaction patterns should be validated in larger cohorts. In addition, non-significant interaction findings should not be interpreted as evidence of the absence of sex-specific differences.

## Conclusion

Sex-related differences in postoperative complications after PE are not uniform and appear to emerge under highly invasive surgical conditions.

## Supplementary Information

Below is the link to the electronic supplementary material.

Supplementary Material 1
