# Supplementary material for: Sex-related differences in postoperative complications after pelvic exenteration and their relationship with surgical invasiveness in a multicenter study
Source: Surg Today. 2026 Jul 15;56(11):2726–35. doi: 10.1007/s00595-026-03415-9 (PMC13722729; doi:10.1007/s00595-026-03415-9)
Supplement: Supplementary file 1 [file 595_2026_3415_MOESM1_ESM.docx]

Supplementary Table 1.

| Variable | Contrast/ Unit | Male  OR (95% CI) | Female  OR (95% CI) | Ratio of ORs  OR (95% CI) |
| --- | --- | --- | --- | --- |
| Age | Per 10-year increase | 1.02 (0.76-1.36) | 2.39 (1.25-4.59) | 0.42 (0.21-0.87) |
| BMI | Per 1 kg/m2 increase | 1.04 (0.94-1.16) | 0.90 (0.74-1.11) | 1.15 (0.91-1.46) |
| ASA-PS | >=3 vs <=2 | 0.96 (0.35-2.62) | 0.26 (0.02-3.14) | 3.69 (0.25-54.17) |
| Cancer type | Recurrent vs primary | 2.10 (0.76-5.84) | 0.71 (0.20-2.58) | 2.94 (0.57-15.19) |
| Distant metastasis | Yes vs no | 1.32 (0.50-3.49) | 1.55 (0.26-9.18) | 0.85 (0.11-6.47) |
| Preoperative therapy | Yes vs no | 1.87 (0.95-3.69) | 2.16 (0.59-7.90) | 0.87 (0.20-3.75) |
| Surgery type | TPE vs APE/PPE | 1.25 (0.57-2.72) | 0.82 (0.19-3.56) | 1.51 (0.29-7.94) |
| Surgical approach | Open vs MIS | 2.09 (0.93-4.71) | 0.21 (0.05-0.85) | 10.02 (1.97-50.88) |
| Anal preservation | Yes vs no | 0.72 (0.36-1.44) | 0.64 (0.18-2.30) | 1.12 (0.26-4.79) |
| Combined bony pelvic excision | Yes vs no | 5.55 (0.71-43.44) | 1.55 (0.26-9.17) | 3.59 (0.24-54.47) |
| Perineal reconstruction | Yes vs no | 1.07 (0.53-2.15) | 1.83 (0.51-6.62) | 0.59 (0.14-2.53) |
| Operative time | Per 60-minute increase | 1.18 (1.04-1.33) | 1.14 (0.93-1.39) | 1.04 (0.82-1.32) |
| Blood loss | Per 100-mL increase | 1.04 (1.01-1.08) | 0.97 (0.90-1.04) | 1.08 (0.99-1.17) |
| Transfusion | Yes vs no | 1.51 (0.77-2.99) | 0.79 (0.21-2.95) | 1.91 (0.44-8.36) |

BMI, body mass index; IQR, interquartile range; ASA, American Society of Anesthesiologists; TPE, total pelvic exenteration; APE, anterior pelvic exenteration; PPE, posterior pelvic exenteration; OP, odds ratio; 95% CI, 95% Confidence interval; ORs in women and men represent the association between each factor and postoperative complications within each sex. The ratio of ORs represents the interaction effect, calculated as the OR in men divided by the OR in women. For categorical variables, contrasts are expressed as the first category relative to the second category.

Supplementary Table 2.

| Variable | Contrast/ Unit | Male  OR (95% CI) | Female  OR (95% CI) | Ratio of ORs  OR (95% CI) |
| --- | --- | --- | --- | --- |
| Age | Per 10-year increase | 0.78 (0.59-1.04) | 2.07 (1.09-3.94) | 0.38 (0.19-0.76) |
| BMI | Per 1 kg/m2 increase | 1.05 (0.95-1.16) | 0.91 (0.72-1.14) | 1.15 (0.89-1.48) |
| ASA-PS | >=3 vs <=2 | 1.27 (0.50-3.24) | 1.23 (0.10-14.96) | 1.04 (0.07-14.97) |
| Cancer type | Recurrent vs primary | 1.73 (0.78-3.84) | 1.01 (0.26-3.96) | 1.71 (0.35-8.31) |
| Distant metastasis | Yes vs no | 0.72 (0.29-1.82) | 2.08 (0.39-11.18) | 0.35 (0.05-2.36) |
| Preoperative therapy | Yes vs no | 1.02 (0.53-1.97) | 1.62 (0.40-6.63) | 0.63 (0.13-2.97) |
| Surgery type | TPE vs APE/PPE | 1.10 (0.51-2.37) | 0.53 (0.09-2.94) | 2.10 (0.32-13.85) |
| Surgical approach | Open vs MIS | 1.07 (0.54-2.14) | 0.63 (0.14-2.88) | 1.69 (0.32-8.94) |
| Anal preservation | Yes vs no | 0.51 (0.25-1.05) | 0.47 (0.11-1.90) | 1.09 (0.23-5.30) |
| Combined bony pelvic excision | Yes vs no | 5.04 (1.63-15.56) | 2.08 (0.39-11.17) | 2.42 (0.32-18.29) |
| Perineal reconstruction | Yes vs no | 1.53 (0.77-3.05) | 1.87 (0.46-7.59) | 0.82 (0.17-3.92) |
| Operative time | Per 60-minute increase | 1.19 (1.07-1.32) | 0.86 (0.69-1.08) | 1.37 (1.07-1.76) |
| Blood loss | Per 100-mL increase | 1.01 (0.99-1.03) | 0.93 (0.84-1.04) | 1.08 (0.97-1.21) |
| Transfusion | Yes vs no | 1.04 (0.55-1.96) | 0.47 (0.11-2.12) | 2.20 (0.43-11.24) |

BMI, body mass index; IQR, interquartile range; ASA, American Society of Anesthesiologists; TPE, total pelvic exenteration; APE, anterior pelvic exenteration; PPE, posterior pelvic exenteration; OP, odds ratio; 95% CI, 95% Confidence interval; ORs in women and men represent the association between each factor and postoperative complications within each sex. The ratio of ORs represents the interaction effect, calculated as the OR in men divided by the OR in women. For categorical variables, contrasts are expressed as the first category relative to the second category.
